# The Protective Role of Cognitive Reserve: A Preliminary Study on Parkinsonian Patients Undergoing Deep Brain Stimulation

**DOI:** 10.3390/jcm13154578

**Published:** 2024-08-05

**Authors:** Eleonora Zirone, Fabiana Ruggiero, Maria Takeko Molisso, Roberta Ferrucci, Angelica De Sandi, Angelica Marfoli, Denise Mellace, Filippo Cogiamanian, Linda Borellini, Enrico Mailland, Elena Pirola, Antonella Ampollini, Marco Locatelli, Sergio Barbieri, Francesca Mameli

**Affiliations:** 1 Foundation IRCCS Ca’ Granda Ospedale Maggiore Policlinico, 20122 Milan, Italy; eleonora.zirone@policlinico.mi.it (E.Z.); fabiana.ruggiero@policlinico.mi.it (F.R.); maria.molisso@policlinico.mi.it (M.T.M.); roberta.ferrucci@unimi.it (R.F.); angelicadesandi@policlinico.mi.it (A.D.S.); filippo.cogiamanian@policlinico.mi.it (F.C.); linda.borellini@policlinico.mi.it (L.B.); enrico.mailland@policlinico.mi.it (E.M.); elena.pirola@policlinico.mi.it (E.P.); antonella.ampollini@policlinico.mi.it (A.A.); marco.locatelli@policlinico.mi.it (M.L.); sergio.barbieri@policlinico.mi.it (S.B.); 2 Department of Oncology and Haemato-Oncology, University of Milan, 20122 Milan, Italy; angelica.marfoli@unimi.it (A.M.); denise.mellace@unimi.it (D.M.); 3 Department of Pathophysiology and Transplantation, University of Milan, 20122 Milan, Italy

**Keywords:** Parkinson’s disease, deep brain stimulation, subthalamic nucleus, cognitive reserve, cognitive outcomes

## Abstract

**Background/Objectives**: High cognitive reserve (CR) has been shown to have beneficial effects on global cognition, cognitive decline, and risk of dementia in Parkinson’s disease (PD). We evaluated the influence of CR on the long-term cognitive outcomes of patients with PD who underwent subthalamic nucleus deep brain stimulation (STN-DBS). **Methods:** Twenty-five patients with PD underwent neuropsychological screening using the Montreal Cognitive Assessment (MoCA) at baseline, 1 year, and 5 years after bilateral STN-DBS. CR was assessed using the Cognitive Reserve Index questionnaire. According to CR score, patients were assigned to two different groups (LowCR group ≤ 130, HighCR group > 130). **Results:** Our data showed that patients in the HighCR group obtained a better performance with the MoCA total score at long-term follow-up compared to those in the LowCR group ([mean ± SE] LowCR group: 21.4 ± 1.2 vs. HighCR group: 24.5 ± 1.3, *p* = 0.05). The cognitive profile of the HighCR group remained unchanged over time. Conversely, the LowCR group had worse global cognition 5 years after surgery (T0: 25.3 ± 0.6 vs. T2: 21.4 ± 1.2, *p* = 0.02). Cognitive decline was not associated with mood, demographics, or clinical variables. **Conclusions:** These preliminary findings suggest that higher CR may be protective in PD cognition after STN-DBS. Specifically, a high CR may help cope with long-term decline in the context of surgical treatment. Quantifying a patient’s CR could lead to more personalized medical care, tailoring postoperative support and monitoring for those at higher risk of cognitive decline.

## 1. Introduction

The cognitive reserve (CR) construct has been proposed to explain the common clinical observation that brain damage of the same magnitude can lead to different levels of cognitive impairment in different people [1]. According to the concept of CR, the brain actively copes with brain damage using pre-existing cognitive processes and compensatory mechanisms. Thus, individuals with high CR can withstand more disease-related pathologies effectively and flexibly, using cognitive paradigms or compensatory brain networks. Differences in CR may result from lifetime experiences, especially educational attainment, intellectual involvement in working activities, and engagement in cognitively stimulating activities during leisure time [2]. 

Since CR can be applied to most conditions in which there is a functional brain change, it becomes crucial in neurodegenerative diseases, such as Parkinson’s disease (PD), where cognitive impairment is a non-motor disturbance common even at an early stage. A decline in the cognitive profile manifests mainly as abnormalities of executive functions; however, visuospatial, memory, and language deficits are also present, and in advanced disease stages, dementia is highly prevalent [3]. 

High CR is associated with mild motor and cognitive deficits in PD. Previous studies on CR and cognition, although mainly using only educational level as a proxy for CR, have suggested that greater CR is related to better global cognition, executive function, attention, memory, and visuospatial function [4,5,6,7].

Subthalamic nucleus deep brain stimulation (STN-DBS) is an effective surgical treatment to improve motor symptoms in people with advanced PD without dementia or psychiatric conditions [8,9]. Although empirical evidence has shown no significant changes in global cognitive function post surgery, a consistent decline in frontal executive function, especially deficits in verbal fluency and inhibition, has been reported in both short-term and long-term follow-up studies [8,10,11]. Despite the importance of CR in influencing cognitive outcomes in neurodegenerative diseases, there is limited research specifically exploring its role in patients with PD undergoing DBS. Two neurostimulation studies addressed the role of CR in patients with PD, but they primarily used educational attainment as a proxy for CR and focused predominantly on its effects on motor function [12,13]. Their preliminary findings collectively suggested that patients with higher educational levels exhibited enhancements in UPDRS-III motor scores [12] and Dual-Task gait performance [13] post surgery compared to those with lower educational backgrounds. Notably, no previous studies have comprehensively assessed how CR specifically influences cognition in this clinical population.

This study aimed to evaluate the impact of CR, comprehensively assessed using the Cognitive Reserve Index questionnaire (CRIq), on the long-term cognitive outcomes of patients with PD undergoing bilateral STN-DBS surgery.

## 2. Materials and Methods

### 2.1. Patients

Twenty-five patients diagnosed with PD who underwent bilateral STN-DBS between 2017 and 2019 were enrolled in this study. All patients were screened at the Center for Movement Disorders of the Foundation IRCCS Ca’ Granda Ospedale Maggiore Policlinico of Milan, Italy, according to the Core Assessment Program for Surgical Interventional Therapies in PD guidelines (CAPSIT-PD) [9]. The CAPSIT-PD cognitive guidelines outline specific inclusion criteria for patients to be considered suitable candidates for surgical intervention and provide for the absence of significant cognitive impairment or dementia as well as severe untreated psychiatric disorders, such as uncontrolled depression or psychosis. 

The following demographic and clinical data were collected at baseline: gender, educational level, age, disease duration at DBS surgery, side of onset, motor phenotype (akinetic-rigid vs. tremor-dominant subtype), Movement Disorder Society-UPDRS Part III scores in ON- and OFF-medication conditions, and levodopa equivalent daily doses calculated for all antiparkinsonian drugs. Ethical approval was obtained from the Ethics Committee (protocol no. 1073_2021), and all participants provided written informed consent.

### 2.2. Surgical Procedures

Surgical procedures were performed by a PD-dedicated neurosurgical team of the Center for Movement Disorders of the Foundation IRCCS Ca’ Granda Ospedale Maggiore Policlinico of Milan. The procedure performed in all patients consisted of the two-stage procedure described by Levi et al. [14]. In the first stage, bilateral intracerebral leads were implanted under local anesthesia in awake patients. Bilateral STN targeting and trajectories were elaborated on preoperative magnetic resonance imaging (MRI) fluid-attenuated inversion recovery, volumetric T2- and T1-weighted sequences with gadolinium. A stereotactic computed tomography scan, performed immediately before surgery with a Cosman-Roberts-Wells stereotactic frame (Radionics [Integra, Plainsboro, NJ, USA]) placed on the head of the patient, was combined with MRI in a dedicated workstation (Stereotaxy, Brainlab, Kapellenstr, Germany). The recording microelectrodes (FHC Inc., Bowdoin, ME, USA) were placed, and their position was tested by recording neurophysiological activity. Final leads (Model 3389 [Medtronic Inc., Minneapolis, MN, USA]) were subsequently implanted. The second stage was performed under general anesthesia and consisted of the placement of a lead extender and an implantable pulse generator. Patients were subsequently discharged, and the stimulation was turned on three to six weeks later, during a second hospitalization.

### 2.3. Clinical Assessment

Neuropsychological screening was performed under the best medical treatment (i.e., in the “medication-ON” condition before DBS, and the “medication-ON” and “stimulation-ON” condition after DBS). Patients were evaluated before surgery (T0), 1 year (T1), and 5 years (T2) after surgery.

The Montreal Cognitive Assessment (MoCA) assessed cognitive profiles at each time point. This comprehensive cognitive screening tool is widely recognized for its ability to detect mild cognitive impairment in patients with neurodegenerative illnesses—a category that includes Alzheimer’s disease, PD, dementia with Lewy bodies, and frontotemporal dementia [15]. We collected participants’ total MoCA scores and domain subscores to explore the following cognitive domains: memory, visuospatial abilities, executive functions, attention, language, and temporal and spatial orientation. The memory domain includes tasks such as word recall and short-term memory retention, while visuospatial abilities are evaluated through tasks involving drawing and understanding spatial relationships. Executive functions are assessed through tasks requiring planning, problem-solving, and abstract thinking. Attention is gauged through sustained focus and concentration tasks. The language domain is tested via naming, repetition, and verbal fluency tasks, and temporal and spatial orientation are evaluated through questions related to the current date, location, and context. It assesses seven areas of cognition for a total possible score of 30 points; a score of <15.51 or less is indicative of cognitive impairment [16].

To exclude the long-term impact of mood on cognition, we administered the Beck Depression Inventory-II (BDI-II) at baseline and at 5 years. The BDI-II is a widely used and validated self-report tool designed to measure the presence and severity of depressive symptoms. The total score is obtained by summing the scores for all 21 items, and it can range from 0 to 63. Generally, BDI-II scores are interpreted as follows: (i) minimal or no depression (0–13 points); (ii) mild depression (14–19 points); (iii) moderate depression (20–28 points); (iv) severe depression (29–63 points). This tool has demonstrated robust psychometric properties, making it an effective screening instrument for depression in the PD population. While it includes somatic elements that may elevate scores in PD patients, these factors do not significantly reduce its ability to differentiate between depressed and non-depressed patients [17].

The CR was evaluated at baseline using the CRIq, a composite tool designed to assess various aspects of CR through the following measures:

(i) Education: This section assesses formal education, including the number of years of schooling and higher education degrees obtained. It considers the level and duration of formal education as a significant contributor to CR.
(ii) Working Activity: This domain evaluates the complexity and duration of the individual’s occupational activities. It considers both paid and unpaid work, emphasizing roles that require problem-solving, decision-making, and other cognitively demanding tasks.
(iii) Leisure Time Activities: This part looks at engagement in activities outside of formal education and work, such as hobbies, social interactions, physical exercise, and other intellectually stimulating pursuits. The variety and frequency of these activities are considered important for building CR [2].

The overall CRIq captures the extent and quality of intellectual engagement and life experiences, providing a comprehensive evaluation of an individual’s CR. 

In our study, patients were assigned to two different groups according to their CRIq score at the time of surgery: low score ≤ 130 (LowCR group) and high score > 130 (HighCR group).

### 2.4. Statistical Analysis

Data were collected in Microsoft Excel, and statistical analysis was carried out using R 4.3.0 (R Core Team Software, Vienna, Austria). The normality of the data was assessed using the Shapiro–Wilk normality test. Continuous variables are reported as mean and standard error, whereas categorical variables are reported as frequency and percentage. Fisher’s exact test for categorical variables and the Wilcoxon rank-sum test for continuous variables were applied to investigate group differences in overall cognitive ability, demographics, and clinical data at each time point. The Friedman and post hoc pairwise Wilcoxon rank-sum tests with Bonferroni correction were used to assess the cognitive performance over time in each patient group. Univariate linear regression analysis was performed for cognitive domains that showed significant changes over time to understand the influence of demographic and clinical variables. Statistical significance was set at *p* ≤ 0.05.

## 3. Results

Twenty-five patients with PD (eleven female; [mean ± SE] age: 58.5 ± 1.7 years; disease duration: 11.3 ± 0.6 years) reported a CRIq total score of 124.8 ± 4.3 (mean ± SE). The demographic and clinical characteristics of the participants are summarized in Table 1. At enrollment, no patients showed signs of cognitive deterioration or severe depression ([mean ± SE] MoCA adjusted score of 25.4 ± 0.5; BDI-II score: 11.3 ± 1.2) according to CAPSIT-PD guidelines [9]. Five years after surgery, an MoCA adjusted score of 22.7 ± 0.9 and no severe deflection of mood (BDI-II score: 9.8 ± 1.6) were observed. Mood was not evaluated in five patients because had a diagnosis of dementia (n = 3) or were unavailable for compilation (n = 2).

### 3.1. LowCR Group vs. HighCR Group

**Figure 1:**
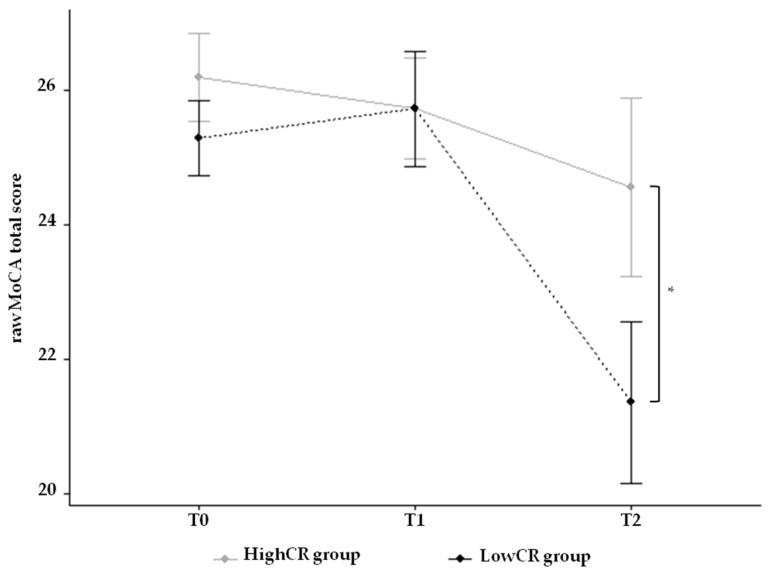
Changes in total MoCA score between HighCR and LowCR groups over time (T0, T1, T2). Asterisk denote statistically significant differences at long-term follow-up (T2). Data are presented as mean and SE.

Baseline demographics and clinical variables were similar between the groups, except for education ([mean ± SE] LowCR group: 11.2 ± 0.9 years; HighCR group: 14.5 ± 1 years; *p* = 0.03) (see Table 1). Comparing the cognitive performance of groups, the data showed that patients with high CR had better performance in the executive domain at baseline (LowCR group: 3.3 ± 0.2; HighCR group: 3.7 ± 0.2; *p* = 0.05). At long-term follow-up, patients with high CR showed better performance in the raw MoCA total score (LowCR group: 21.4 ± 1.2; HighCR group: 24.5 ± 1.3; *p* = 0.05) (see Figure 1).

### 3.2. LowCR Group

Fourteen patients (seven female; [mean ± SE] age: 57.1 ± 2.7 years; disease duration: 10.3 ± 0.7 years) were included in the group with CRIq ≤ 130 (108.1 ± 2.8) (see Table 1). 

In this group, the MoCA total score decreased at T2 (raw score: 21.4 ± 1.2) compared to T0 (raw score: 25.3 ± 0.6; *p* = 0.02) and T1 (raw score: 25.7 ± 0.9; *p* = 0.004). Similarly, patients showed lower memory domain subscore in T2 than in T1 (T1: 3.1 ± 0.4; T2: 2.2 ± 0.3; *p* = 0.03). Language domain subscore was also significantly lower at 5 years (4.3 ± 0.3) compared to baseline (5.6 ± 0.1; *p* = 0.03) and at 1 year after surgery (5.6 ± 0.2; *p* = 0.02). No changes in mood were observed over time (BDI-II scores: T0: 11.1 ± 1.4; T2: 11.5 ± 2.8; *p* = 0.81). Univariate linear regression analysis showed no significant relationship between clinical and demographic data, total MoCA score at T2, and language. The left onset side predicted a better memory performance 5 years after surgery (β = 1.7; 95% CI = 0.41–2.99; *p* = 0.01).

### 3.3. HighCR Group

Eleven patients (four female; [mean ± SE] age: 60.2 ± 1.7 years; disease duration: 12.5 ± 0.9 years) had a total CRIq score > 130 (146.1 ± 2.9) (see Table 1). No significant changes in the overall cognitive ability or mood over time were found in this group of patients. 

## 4. Discussion

This study aimed to explore CR as a protective factor against long-term cognitive outcomes in patients with PD who underwent bilateral STN-DBS surgery. Identifying factors influencing the long-term cognitive outcomes of DBS treatment in this clinical population is a relevant field of investigation, paving the way for personalized treatment with the potential to maximize effectiveness. 

Our preliminary findings suggest that a higher CR may be protective in PD cognition after STN-DBS. Preoperatively, total cognitive scores and domain subscores were similar between groups with high CR and low CR, except for the executive domain in which, according to previous studies using educational level, premorbid IQ, and the CRIq itself as CR proxies, patients with higher CR showed significantly better performance [4,5,6,7]. However, after surgery, the cognitive profiles of the two groups showed a different evolution; only patients with a high CR remained unchanged over time, while patients with a lower CR worsened at 5 years of follow-up. Our results seem to confirm the data of Hindle’s longitudinal cohort study [18], which showed that in patients with PD with normal cognition at baseline, as well as in our sample, a higher education level was associated with better global cognition after 4 years. Therefore, our observations suggest that high CR slows cognitive decline in PD even in surgical treatment such as DBS. 

Interestingly, this worsening in cognitive profile in patients with low CR was not correlated with the age of the patient, duration of the disease, motor phenotype, severity of the symptoms, or mood disorders; therefore, we suggest that CR may have had an impact on cognitive evolution in our sample of patients. 

Furthermore, unlike the high CR group, the low CR group showed a specific worsening in the memory and language domains 5 years after DBS. Given that deficits in these domains are considered risk factors for a more rapid progression toward dementia [3], our results seem to confirm that CR can be a protective factor against cognitive decline, even in patients with PD. According to previous studies conducted mainly on patients with Alzheimer’s disease, CR raises the tolerance threshold for cognitive impairment and delays the clinical manifestations of decline. Most likely, a high CR does not protect individuals from developing neurodegenerative diseases but could mitigate the impact of pathology on the clinical expression of dementia [19]. 

Finally, in the long-term follow-up, we observed that patients with a high CR score also showed a mild impact on the executive domain; however, this was not significant compared to their baseline performance, which deserves to be explored in more detail in future studies with a larger sample of patients.

Despite the insights obtained from this study, caution is required for the following reasons. First, our population was recruited only from a clinical center to which patients from the same sociocultural background had access, and our sample showed an overall high CRIq score at baseline; therefore, it may not be representative of the general population. Second, we explored cognitive domains exclusively with the MoCA. A more detailed neuropsychological assessment would allow for better characterization of PD’s cognitive profile and accurate analyses of the role of CR. Third, the small sample size limits the generalizability of our data, and a large multicenter study is required to further examine this issue in patients with PD who are undergoing DBS. Finally, this study did not include other clinical variables that could affect cognitive outcomes, such as cerebrovascular risk, white matter lesions, genetic mutations, imaging data, and biomarkers.

Future research should expand its focus by including larger patient cohorts to comprehensively examine the impact of various clinical variables, structural neuropathological changes, genetic factors, and surgical interventions on the evolution of cognitive profiles associated with CR. This broader approach will enhance our understanding of how these factors interact and influence cognitive outcomes in neurodegenerative conditions such as PD, especially in the context of invasive treatments like DBS.

## 5. Conclusions

In conclusion, our preliminary findings on the cognition of patients with PD undergoing STN-DBS suggest that high CR may help them cope with long-term decline after surgery. 

These findings have several implications for clinical practice and future research. Overall, this study underscores the protective role of CR in maintaining long-term cognitive function, particularly after surgery. The results suggest that patients with higher CR are better equipped to handle the cognitive stresses associated with surgical interventions, while those with lower CR are at greater risk of cognitive decline. This highlights the need for targeted strategies to boost CR in vulnerable populations. An accurate assessment of the CR of patients undergoing surgery would allow us to predict the evolution of the long-term cognitive profile leading to more personalized medical care, adapting postoperative support and monitoring of patients at greater risk of cognitive decline.

Further studies are needed to investigate how CR modulates cognitive decline in patients with PD and frontal-subcortical disorders, which are among the most frequently occurring adverse events after DBS surgery. Establishing the effective impact of CR on cognitive changes may improve the selection criteria for DBS inclusion and maximize the effectiveness of treatment. 

## Figures and Tables

**Table 1:** Group demographics and clinical characteristics.

Demographics and Clinical Data		All Patients (n = 25)	LowCR Group (n = 14)	HighCR Group (n = 11)	HighCR vs. LowCR Groups
		N (%)	N (%)	N (%)	*p*-Value
Gender	Female	11 (44.0)	7 (50.0)	4 (36.4)	0.69
	Male	14 (56.0)	7 (50.0)	7 (63.6)	
Side of onset	Right	9 (36.0)	4 (28.6)	5 (45.5)	0.43
	Left	16 (64.0)	10 (71.4)	6 (54.5)	
Motor phenotype	Tremor-dominant	14 (56.0)	6 (42.9)	8 (72.7)	0.23
	Akinetic-rigid	11 (44.0)	8 (57.1)	3 (27.3)	
		**Mean ± SE**	**Mean ± SE**	**Mean ± SE**	
Age (years)		58.5 ± 1.7	57.1 ± 2.7	60.2 ± 1.7	0.51
Educational level (years)		12.7 ± 0.7	11.2 ± 0.9	14.5 ± 1	**0.03**
Disease duration (years)		11.3 ± 0.6	10.3 ± 0.7	12.5 ± 0.9	0.14
MDS-UPDRS-III score	Med. ON	17.9 ± 1.4	16.9 ± 1.4	19.1 ± 2.7	0.72
	Med. OFF	38.7 ± 2.1	39.4 ± 2.5	37.8 ± 3.6	0.93
LEDD score		1044.3 ± 74	1079.9 ± 105.9	998.9 ± 104.7	0.89
CRIq score	Education	109.5 ± 3.7	103.9 ± 3.6	116.5 ± 6.7	**0.04**
	Working activity	113.4 ± 4.5	97 ± 2.6	134.3 ± 4.8	**<0.001**
	Leisure time	133 ± 5.2	117.4 ± 4.6	152.8 ± 6.4	**0.001**
	Total score	124.8 ± 4.3	108.1 ± 2.8	146.1 ± 2.9	**<0.001**

Abbreviations: CRIq: Cognitive Reserve Index questionnaire; LEDD: levodopa equivalent daily dose; MDS-UPDRS-III: Movement Disorder Society-Unified Parkinson’s Disease Rating Scale part III; Med: medication; SE: standard error.

## Data Availability

The data presented in this study are available on request from the corresponding author.
